# Selenium in the Treatment of Graves’ Hyperthyroidism and Eye Disease

**DOI:** 10.3389/fendo.2020.608428

**Published:** 2021-01-26

**Authors:** Giulia Lanzolla, Michele Marinò, Claudio Marcocci

**Affiliations:** Department of Clinical and Experimental Medicine, Endocrinology Unit II, University of Pisa and University Hospital of Pisa, Pisa, Italy

**Keywords:** Graves’ hyperthyroidism, Graves’ orbitopathy, oxidative stress, reactive oxygen species, selenium, selenocysteine, selenoproteins

## Abstract

Based on the role of oxidative stress in the pathogenesis of Graves’ hyperthyroidism (GH) and Graves’ Orbitopathy (GO), a therapy with the antioxidant agent selenium has been proposed and a number of studies have been performed, both *in vitro* and *in vivo*. In GH, reactive oxygen species (ROS) contribute to the thyroid and peripheral tissues damage. In GO, tissue hypoxia, as well as ROS, are involved in the typical changes that occur in fibroadipose orbital tissue and the perimysium of extraocular muscles. Antioxidants have been proposed to improve the effects of antithyroid drugs in GH patients, as well as the remodeling of orbital tissues in patients with GO. Here, we reviewed the literature on the possible beneficial effects and clinical use of selenium in the management of patients with GH and GO. A randomized clinical trial on the use of selenium in patients with mild GO provided evidence for a beneficial effect; no data are available on more severe forms of GO. Although the real effectiveness of selenium in patients with GH remains questionable, its use in the management of mild GO is generally believed to be beneficial, and selenium administration has been included in the clinical practice for the patients with mild eye disease.

## Introduction

Selenium has been proposed for the management of thyroid diseases, including Graves’ disease (GD), Graves’ Orbitopathy (GO), and chronic autoimmune thyroiditis (1–7). The efficacy of selenium in GD and GO is based on its anti-oxidant properties, being oxidative stress involved in the pathogenesis of both conditions (8–12).

GD is an autoimmune disease with a prevalence of ~1% (13), which affects mainly the thyroid. Extrathyroidal manifestations, including GO, pretibial myxedema, and acropachy, can be observed to various extents (13–17). The major pathogenetic mechanism of GD is the stimulation of the thyroid stimulating hormone receptor (TSH-R) by autoantibodies that bind to it and promote thyrocyte proliferation and activity, resulting in thyroid hyperfunction (13). The symptoms of thyrotoxicosis are often nonspecific, so patients with GD may present in various ways. Heat intolerance, tachycardia, inappropriate feelings of anxiety and apprehension, hyperactivity and weight loss are common. A diffuse goiter can be visible or palpable, with a systolic phase bruit found over it. Systolic blood pressure may be elevated, and hepatomegaly or splenomegaly may be observed (16). The complex pathogenetic interplay of GD includes, among others, oxidative stress (16–18), because of which selenium and other antioxidant agents have been proposed for the management of Graves’ hyperthyroidism (GH). In this paper we review the role of oxidative stress in GH, as well as the most significant studies on the use of selenium in the management of patients with GH and GO. The molecular mechanisms by which the disruption of the cell redox state plays a role in the pathogenesis of GO, as well as the potential beneficial effect of other antioxidant agents in patients with GO, are largely discussed in another review article in this issue of the journal.

## Cell Redox State and Evaluation of Oxidative Stress

The balancing of the cell redox state is a key point in cellular homeostasis. Reactive oxygen species (ROS), including hydroxyl radicals (OH^-^), hydrogen peroxide (H_2_O_2_), superoxide anions (O^2^), and lipid peroxides, are molecules highly reactive because of the presence of unpaired electrons (19, 20). The increase in ROS production, as well as the decrease in their elimination, can disrupt the balance of the cell redox state, resulting in oxidative stress and breaking of cellular homeostasis (19, 20). As oxidizing agents, ROS interfere with intracellular functions, being capable of damaging various cellular elements, including cell membranes, proteins, lipids and nucleic acids, and ultimately resulting in mitochondrial dysfunction and loss of enzymatic activity (19, 20). Under physiological conditions, antioxidant agents, namely glutathione (GSH), superoxide dismutase (SOD), glutathione peroxidase (GPX) and catalase, act as ROS antagonists, therefore contributing to the maintenance of the cell redox state (21).

The oxidant/antioxidant cell balance can by evaluated by several plasma and/or erythrocyte markers (20, 21). In healthy subjects, markers of oxidative stress, as well as of the antioxidant system, are lowly represented. The induction of an oxidative stress state promotes two different reactions in the cells, depending on how long it lasts. Initially, an increase in oxidative markers is accompanied by an increase in antioxidant markers, the latter being a homeostatic response aimed at avoiding cell damage. A long-lasting state of oxidative stress is however characterized by low levels of antioxidant markers, because of exhaustion of the antioxidant cell systems (20, 21).

## Oxidative Stress in GH

Thyrotoxicosis is a hypermetabolic state characterized by high consumption of intracellular ATP and oxygen, and by dysfunction of the mitochondrial respiratory chain, which leads to saturation of the physiological antioxidant systems, resulting in an uncontrolled production of ROS in peripheral tissues as well as in the thyroid (22–25). Studies in thyrotoxic rats suggest that the oxidative stress may be involved in the pathogenesis of thyrotoxic myopathy and cardiomyopathy (26) and *in vitro* studies have shown that exposure of thyroid cell to H_2_O_2_, particularly when made selenium-deficient, induces DNA double strand breaks, apoptosis, necrosis and mutagenesis (24). On this basis, we might speculate that the oxidative stress might exert a dual action in patients with GH. The ROS-induced damage of thyroid epithelial cells could lead to an increased release of autoantigens and production of TSH-R autoantibodies (TRAb), and the peripheral tissue damage might contribute the clinical manifestations of hyperthyroidism.

### Animal Studies

Studies in animal models suggest that thyrotoxicosis, induced by the administration of triiodothyronine (T3) or thyroxine (T4), promotes oxidative stress as well as the response of the physiological antioxidant machinery (22–25). It has been demonstrated that MDA, is significantly higher in thyrotoxic than in control and euthyroid rats (25, 26). Moreover, the oxidative stress induced by thyrotoxicosis promotes an increase in erythrocyte antioxidant parameters, namely SOD and GPX. Interestingly, the administration of Vitamin E, which has antioxidant properties, reduces MDA, SOD and GPX in hyperthyroid rats (26), and it seems also to be protective against a thyroxine-induced increase of lipid peroxidation in cardiac and skeletal muscles (27).

### Human Studies

The most relevant clinical studies on the role of oxidative stress in thyroid diseases have been performed in GH patients, either with uncontrolled hyperthyroidism, or after restoration of euthyroidism with antithyroid drugs (ATD) or radioiodine (28–33). It has been reported that the levels of oxidative stress markers, including hydrogen, lipid peroxides, MDA and thiobarbituric acid-reacting substances (TBARS) in the serum, plasma and erythrocytes of hyperthyroid patients were higher than in euthyroid subjects (28–33). Moreover, a correlation between serum thyroid hormones and lipid peroxidation products was observed in hyperthyroid patients (28–33). These findings do not seem to be specific for GH, but rather for hyperthyroidism in general, having been observed also in patients with subclinical hyperthyroidism due to multinodular goiter (31). The evaluation of the antioxidant defense system in thyrotoxic patients resulted in conflicting findings (10, 28, 29, 33). Komosinska-Vassev et al. reported higher levels of SOD, CAT and GPX in the erythrocytes of GH patients than in age-matched controls. However, no differences in serum glutathione reductase (an antioxidant enzyme involved in GSH synthesis) and in the total antioxidant status were found, likely suggesting rapid exhaustion of the antioxidant system (10). Bednarek et al. reported an increase in plasma SOD and catalase in patients with GH of short duration (1–2 months) compared with healthy subjects, but not of GPX and glutathione reductase, which were, conversely, decreased (29). On the contrary, Aslan et al. reported a significant reduction of the total antioxidant activity in 36 hyperthyroid patients with an average duration of hyperthyroidism of 2.3 ± 1.5 months compared with controls (33). Finally, a study involving 69 GD patients with hyperthyroidism lasting more than 6 months showed decreased levels of erythrocyte SOD and catalase activities compared with controls, with no difference in erythrocyte GPX and total antioxidant activities (28). The duration of the hyperthyroid status at the time of the evaluation can explain, at least in part, the conflicting data on antioxidant activity in patients with GD. Probably, patients with hyperthyroidism of longer duration have exhausted the antioxidant defense system, which leads to the reduction of the antioxidant capacity (34, 35). It has been also reported that the monoclonal thyroid stimulating antibody M22 as well as polyclonal serum thyroid stimulating antibodies (TSAbs) from patients with GH, promote ROS generation and lipid peroxidation in HEK cells that stably overexpressed the human TSHR (HEK-293 TSHR) (36). ATD reduce the levels of oxidative stress markers, thereby improving the activity of the intra- and extracellular antioxidant defense systems, due to restoration of euthyroidism and possibly to their antioxidant properties (28–30, 32). As discussed in another review on the same issue of this journal, oxidative stress also plays a role in GO, and the beneficial effect of antioxidant agents are supported by *in vitro* and human studies (1–6, 11, 37–46).

## Selenium

Selenium is a trace mineral which acts following incorporation as selenocysteine into selenoproteins, among which thioredoxin reductases (TRs), GPX, and iodothyronine deiodinases (D1, D2 or D3) are the best known (3–5, 47). In this regard, D1-3 are homologous proteins consisting of 250-300 amino acids, with a single transmembrane domain located at the N-terminus. They are involved in the reductive deiodination of thyroid hormones. The most remarkable feature of all three deiodinases is the presence of a selenocysteine residue in the center of the amino acid sequence. D1, encoded by DIO1 gene, is predominantly expressed in the liver, kidney and thyroid. It catalyzes the outer and inner ring deiodination of iodothyronine derivatives, with a preference for reverse T3 (rT3) and iodothyronine sulfates. D2, encoded by DIO2 gene, is primarily expressed in the thyroid, brain, anterior pituitary and brown adipose tissue, where contributes local conversion of T4 in T3. Moreover, the pituitary enzyme is involved in the negative feedback regulation of TSH and TRH secretion. The DIO3 gene codes for D3, which is detected in brain, skin, liver, intestine, placenta and pregnant uterus, where it catalyzes the inactivation of T4 and T3. Since D1-3 are selenoproteins, selenium deficiency would be expected to result in their reduced activities in different tissues, although this effect is observed only for D1 in the liver and kidney and not for D2 and D3 in other tissues (48).

Selenoproteins have antioxidant and enzymatic capacity (47) and, in the thyroid, where they are highly expressed, influence the balance of the cell reduction-oxidation activities (49). Furthermore, selenoproteins are essential for activated T-cell function, being involved in the proliferation of T-cells in response to T-cell receptor stimulation. It has been also reported that selenium supplementation promotes differentiation of CD4+ T cells into T-helper-1 (Th1) rather than T-helper-2 (Th2) effector. Among the immunomodulating functions, selenoproteins increased cytotoxic lymphocyte-mediated tumour cytotoxicity and natural killer activity (50). It has been also reported that selenium may regulate the inflammatory response by reducing the release of tumor necrosis factor α (TNFα) and cyclooxygenase (COX2), as well as the activation of nuclear factor κB (NF-κB) which is one of the transcriptional factors involved in the immune and pro-inflammatory response. Elevated selenium levels increase GPX, which inhibits IκB-α phosphorylation and consequently the translocation of NF- κB (51).

Dietary sources of selenium include meat, seafood, shellfish, offal, eggs and cereals (47, 49). The bioavailability of selenium varies depending on the type of food and content in the soil for growing crops and fodder. Furthermore, additional factors, such as selenium speciation, soil pH or the presence of ions complexed with selenium, play a role in the bioavailability of the mineral (47). Selenium dietary intake is followed by absorption in the gastrointestinal tract and transport to the liver, where it is incorporated into selenoglycoproteins containing up to 10 selenium residues per molecule. Selenoglycoproteins finally reach peripheral tissues, where their concentration is proportional to the degree of oxidative stress (47–50).

The measurement of total serum selenium concentration or circulating selenoproteins, including GPX-3 and selenoprotein P, can be used to evaluate the selenium status, which is variable depending on the geographical area, being high in North America and relatively low in most European countries, in particular in the Eastern Europe (50–52). The Office of Dietary Supplements has established that the recommended daily allowance (RDA) is 55 µg in men and women. Selenium intake in several European countries is lower than in the USA, because of its lower content in the soil. Values range from around 30 µg in UK, Germany, Sweden and Slovakia to around 70 in Netherlands and Switzerland (47, 50). To properly interpret these data, we need to have appropriate standards against which to compare them. There is no consensus on this issue. The UK reference nutrient intake of 75 µg per day in men and 60 in women has been determined as the amount needed to maximize the activity of the GPX in plasma (which occurs at a selenium concentration of about 95 µg/L). Current UK intake are about half the value. On the other hand, selenium excess has been suggested to increase the risk of type 2 diabetes and of malignancies (50, 52). However, the issue is still matter of debate and no adverse events for selenium doses not exceeding 200 µg/day were reported by a number of studies, including one in which relatively high levels of selenium were reached with supplementation (~190 µg/L after 90 days of treatment) (3, 50). It is therefore possible that selenium might lead to subclinical alterations, for example on glucose metabolism, in selected patients, with no real clinical impact (1, 4, 53, 54).

In this regard, several trials have shown a benefit of selenium supplementation on the risk of cancer incidence and death, as well as the risk of developing hyperglycemia. However, two large trials performed in USA have shown that these benefits are no longer seen in patients whose baseline serum selenium concentration was above 122 μg/L, in whom, conversely, these risks were increased. On this basis, Rayman proposed that 122 µg/L represents the concentration of baseline serum selenium that “delineates a change in risk, from lower to higher, of developing cancer and type 2 diabetes in patients treated with selenium supplementation of 200 µg per day” (50). This observation may be relevant for individuals living in countries, like the USA, where a large proportion of people has a serum selenium concentration above this cut-off. Therefore, it has been proposed to measure serum selenium concentrations before administering the supplement, in order to avoid overdosage in subjects with baseline serum concentrations higher than 122 µg/L (53). In our opinion, the measurement of serum selenium before supplementation might be useful in individual living in countries with high selenium intake, like the USA, to avoid the risk of potential disadvantages, but not individuals living in countries, like most European countries, where dietary selenium intake is low and high concentrations of serum selenium are very unlikely to be achieved with supplementation.

Selenium supplements contain sodium selenite or selenomethionine, and the main difference between them is that, after saturation of selenoproteins, selenite is excreted whereas selenomethionine can further increase serum selenium through its incorporation into proteins (1, 4). Consequently, the effect of selenite is strictly linked to the individual state of selenium, whereas using selenomethionine as a supplement, the concentration of plasma selenium increases also in subjects with a sufficient selenium concentration to begin with.

## Use of Selenium in GH

Based on the above described role of oxidative stress in GH and the antioxidant activity of selenium, clinical studies were performed to investigate its effect in GH patients. The hypothesis is that selenium deficiency could enhance oxidative stress in thyrotoxicosis, by worsening the antioxidant machinery in response to ROS. To address this issue, a number of studies have tested selenium, either alone or within a mixture containing other antioxidant agents, in GH patients treated with ATD (2, 3, 55–57) (**Table 1**). These investigations did not provide clear-cut results. Vrca et al. performed a study in Croatia, an area characterized by moderate selenium deficiency, randomizing GH patients to therapy with methimazole (MMI) plus an antioxidant mixture containing β-carotene, Vitamin C and Vitamin E and a relatively low dose of selenium (60 μg), or MMI alone (55). The main difference between the two groups was that euthyroidism was more rapidly reached in the former group, likely reducing the period of exposure to oxidative stress. Moreover, a better response to treatment in terms of improvement of LDL-cholesterol levels was found in patients treated with the antioxidant mixture compared with those given MMI alone (55). Similar date on thyroid function were reported by Guerra et al. in patients given a mixture containing low doses of selenium (15 μg/day), compared to MMI alone (57).

**Table 1:** Selenium in Graves’ Hyperthyroidism (GH).

Reference	Journal and years	Type of study	Methods	Dosage of compounds used	Results
Vrca VB. et al. (53)	Acta Pharm. 2012	Prospective, placebo-controlled, clinical trial	Fifty-five patients with newly diagnosed GH were randomized to receive methimazole alone or methimazole plus a fixed combination of antioxidant agents	The mixture of antioxidants contained: **β-carotene** (6 mg), **selenium** (60 μg), **vitamins C** (200 mg) **and E** (36 mg)	**Beneficial effect on GH** Better and faster normalization of thyroid function was observed in patients with GH treated with methimazole plus the mixture of antioxidant agents compared with control group. In addition, a better response to treatment, in terms of improvement of LDL-cholesterol levels, was found in patients treated with the antioxidant mixture compared with those given methimazole alone
Calissendorff J. et al. (2)	Eur Thyroid J 2015	Prospective, placebo-controlled, clinical trial	Thirty-eight consecutive patients with newly diagnosed and untreated GH were randomized to receive “block and replace” treatment with methimazole and levothyroxine, plus Selenium or alone for 9 months	**Selenium** (200 μg daily orally)	**Beneficial effect on GH** Patients treated with selenium had a better control of hyperthyroidism: at 18 weeks, the serum levels of FT4 were lower in the selenium group compared to the placebo (14 vs. 17 pmol/l group, p = 0.01). Similar results were observed also at 36 weeks (15 vs. 18 pmol/l, p = 0.01). In accordance, the TSH levels increased more in the selenium group at 18 weeks (0.05 vs. 0.02 mIU/l, p = 0.04)
Leo M. et al. (3)	J Endocrinol Invest 2017	Prospective, placebo-controlled, clinical trial	Thirty consecutive patients with untreated GH were randomized to receive Methimazole plus Selenium vs methimazole alone for 3 months	**L-seleno-methionine** (166 μg daily orally)	**No beneficial effect on GH**. Administration of Methimazole leads to the normalization of FT3 and FT4, with no difference between groups. Serum levels of malondialdehyde, a marker of oxidative stress, was similarly high in the two groups and decreased significantly after therapy, with no difference between groups. The results suggested that selenium had not significant effect on short-term control of hyperthyroidism
Kahaly GJ. et al. (54)	J Clin Endocrinol Metab. 2017	Prospective, placebo-controlled, clinical trial	Sixty-one consecutive patients with untreated GH were randomized to receive Methimazole plus Selenium or methimazole alone for 6 months	**Sodium** **selenite** (300 μg daily orally)	**No beneficial effect on GH**. The response to treatment, in terms of thyroid function normalization of thyroid hormones, was very similar in the two groups at week 24. During a 12-week follow-up, GH relapsed in 48% of patients included in the selenium group and in 44% of patients of the placebo group. Serum concentrations of Selenium and selenoprotein P were unrelated to response or recurrence rates. At week 36, 12 of 29 patients (41%) and 15 of 33 patients (45%) were responders and still in remission in the selenium and placebo groups, respectively.

Three studies were performed using “pure” selenium rather than a mixture of antioxidants (2, 3, 56) (**Table 1**). In one of these studies (56), hyperthyroid, selenium-deficient patients were randomized to receive a block-and-replace regimen (MMI plus levothyroxine), plus 200 μg/day of selenium or placebo. A slightly better control of hyperthyroidism was observed in the selenium group (56). Two additional randomized clinical trials performed in GH patients treated with MMI alone did not show beneficial effects on thyroid function and peripheral manifestations of hyperthyroidism in patients given selenium compared with placebo (2, 3). The conflicting results between the first study and the subsequent two can be explained, at least in part, by the different levels of baseline selenium, which was insufficient only in the first one. These findings support the main hypothesis of a role of ROS in GH, which might be relevant especially in patients with selenium deficiency. Thus, a supplementation with selenium can be considered in these cases, whereas, in our opinion, there is no sufficient evidence to recommend the routine addition of selenium to ATD in the management of all patients with GH.

## Use of Selenium in GO

Over the last few years, in addition to the most common treatment for the management of moderate-to-severe and active GO, namely high dose oral and intravenous glucocorticoids (GC), orbital irradiation and surgical procedures (58), other medications have been proven effective to various extents, including rituximab (59), the recently FDA-approved teprotumumab (60, 61), mycophenolate (62), and tocilizumab (63). Until recently, the treatment of mild GO was typically limited to local measures (19 but ~15% of patients GO progress to an extent that requires specific treatments (13, 58). In general, major treatments are not recommended for mild GO, unless there is a sufficient impairment in the quality of life, which justifies the risk of GC-related adverse events (58, 64–66). In view of the role of oxidative stress in GO, antioxidant supplements have been proposed as a possible therapeutic approach, and basic and clinical studies have investigated the effect of selenium, due to its antioxidant and immunomodulating actions.

### In Vitro Studies

**Figure 1:**
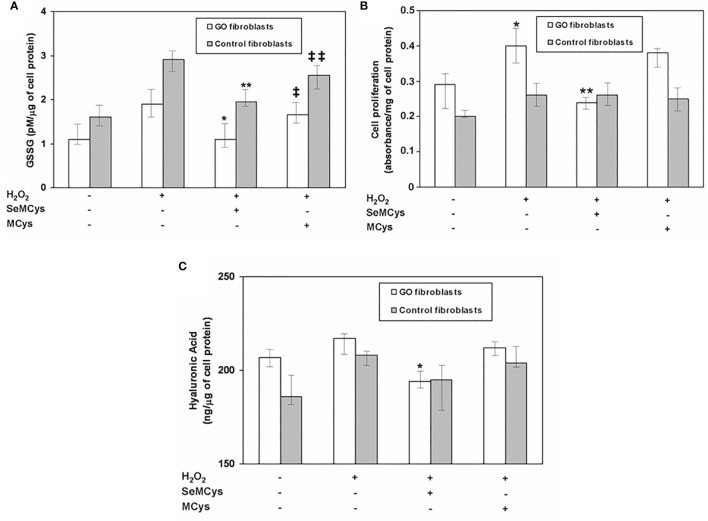
**(A)** Effect of selenium (10 μMol) or of the negative control methylcysteine (MCys) (10 μMol) on Glutathione disulfide (GSSG), released after treatment with H_2_O_2_ (5 μMol), in fibroblasts from patients with Graves’ orbitopathy (GO fibroblasts) or from control patients. P* and **P = 0.02 vs H_2_O_2_; ‡ and ‡‡ P = NS vs H_2_O_2_; P = NS between GO and control fibroblasts; **(B)** Effect of selenium (10 μMol) or methylcysteine (MCys) (10 μMol) on cell proliferation, induced by H_2_O_2_ treatment (5 μMol), in GO and control fibroblasts. *P = 0.02 vs untreated cells; **P = 0.02 vs H_2_O_2_; P = 0.003 between GO and control fibroblasts; **(C)** Effect of selenium (10 μMol) or methylcysteine (MCys) (10 μMol) on hyaluronic acid (HA) release in GO and control fibroblasts. *P = 0.02 vs H_2_O_2_; P = 0.02 between Graves’ Orbitopathy and control fibroblasts. Reprinted with permission by Thyroid 2017, Volume 27, pp. 271–278, published by Mary Ann Liebert, Inc., New Rochelle, NY.

A couple of *in vitro* studies have provided evidence for a beneficial effect of selenium in primary cultures of orbital fibroblasts (OFs) (38, 65, 66) (**Table 2**). In a first study (38), after induction of oxidative stress by treating OFs with H_2_O_2,_ GPX activity, glutathione disulfide (GSSG), cell proliferation, HA and pro-inflammatory cytokines were measured, in the presence or absence of selenium-(Methyl)-selenocysteine (SeMCys). H_2_O_2_ induced oxidative stress in OFs, reflected by a dose-dependent increase in GSSG, a known measure of cell response to ROS (**Figure 1**). SeMCys reduced the effects of H_2_O_2_, providing evidence for an antioxidant action of selenium in OFs. The effect of selenium was observed in OFs from both GO patients and control subjects, which should not be seen as a limitation for its clinical use. Thus, in the same study the authors reported that proliferation of OFs, as well as the production of HA, were significantly greater in GO than in control fibroblasts. Moreover, SeMCys significantly reduced cell proliferation and hyaluronic acid (HA) release in GO OFs, whereas no effects were observed in control OFs (38). These findings might suggest that fibroblasts from GO patients are somehow different, leading to a different response to oxidative stress and selenium activity.

**Table 2:** Selenium in Graves’ Orbitopathy (GO).

Reference	Journal and years	Type of study	Methods	Dosage of compounds used	Results
Rotondo Dottore G. et al. (65)	Thyroid 2016	*In vitro* study	Primary cultures of orbital fibroblasts from 6 GO patients and 6 control subjects were obtained. To induce oxidative stress, cells were incubated for 24 h with medium containing H_2_O_2_ at various concentrations. The primary objective was to assess the effects of selenium in GO fibroblasts.	Cells were pre-incubated for 2 days with medium without compounds or containing **Se-methyl-selenocysteine hydrochloride (SeMCys)** or, as control, **methyl-cysteine** at various concentrations. Cell proliferation, hyaluronic acid (HA) and pro-inflammatory cytokines production were measured	**Selenium reduced proliferation and release of HA and cytokines in GO fibroblasts**. H_2_O_2_ promoted an increased release of glutathione disulfide (GSSG), a marker of oxidative stress, and of fibroblast proliferation, which were reduced by selenium. H_2_O_2_ promoted the production of cytokines involved in GO pathogenesis, namely TNF-α, IL1-β and IFN-γ. The increase in TNF-α and IFN-γ was rescued by selenium. Whereas the effects of selenium were similar in GO and control fibroblasts concerning oxidative stress and cytokines, they were exclusive to GO fibroblasts concerning proliferation and HA production.
Rotondo Dottore G. et al. (66)	Endocrine. 2017		Primary cultures of orbital fibroblasts from 6 patients with long lasting inactive GO and 6 control subjects were obtained. To induce oxidative stress, cells were incubated for 90 min at 37°C with medium containing H_2_O_2_ 50 μM. The aim of the study was to evaluate the effects of selenium in GO fibroblasts.	Cells were preincubated for 2 days at 37°C with medium without compounds containing **SeMCys** or, as control, **methyl-cysteine** (MCys) at a 10 μM concentration. Cell vitality, lactate dehydrogenase production (as a measure of cell necrosis) and apoptosis were measured	**Selenium reduced cell damage in GO fibroblasts** SeMCys rescued from H_2_O_2_-dependent cytotoxicity, by reducing necrosis and apoptosis, with no difference between GO and control fibroblasts. MCys had no effect. To determine whether the findings reflected the antioxidant actions of selenium, the assessment of GSSG was performed. H_2_O_2_ promoted the increased production of GSSG, which was counteracted by SeMCys, but not by MCys, with no differences between GO and control fibroblasts.
Marcocci C. et al. (4)	N Engl J Med 2011	Prospective, multicenter, placebo-controlled clinical trial	One hundred fifty-nine patients with mild GO were randomized to receive sodium selenite or compared with placebo for 6 months	**Sodium selenite** (100 μg twice daily orally), or **pentoxifylline** (600 mg twice daily orally)	**Beneficial effect of selenium in mild GO**. Compared with placebo, Selenium, but not pentoxifylline, leads to the improvement of quality of life (P < 0.001), eye overall outcome (P = 0.01) and slowed the progression of GO (P = 0.01), at the 6 months evaluation.

In a subsequent study, the same authors reported a dual effect of H_2_O_2_ on cell proliferation, depending on dose used (67). At low concentrations, H_2_O_2_ promoted cell proliferation, whereas at high concentration progressively decreased cell vitality and cell proliferation. The effects of both high and low dose H_2_O_2_ were inhibited by SeMCys which, interestingly, inhibited HA synthesis in GO, but not in control fibroblasts, even though H_2_O_2_ did not affect HA release (67). This observation suggests that selenium may influence HA release, at least in part, regardless of the oxidative stress induced by H_2_O_2_, through mechanisms that are still to be clarified. Furthermore, a reduction in the release of pro-inflammatory cytokines induced by low dose H_2_O_2_, namely interferon-γ (IFNγ) and TNFα, was found in both GO and control OFs treated with SeMCys (67, 68), contributing the beneficial effects of selenium in GO OFs.

### Clinical Studies

**Figure 2:**
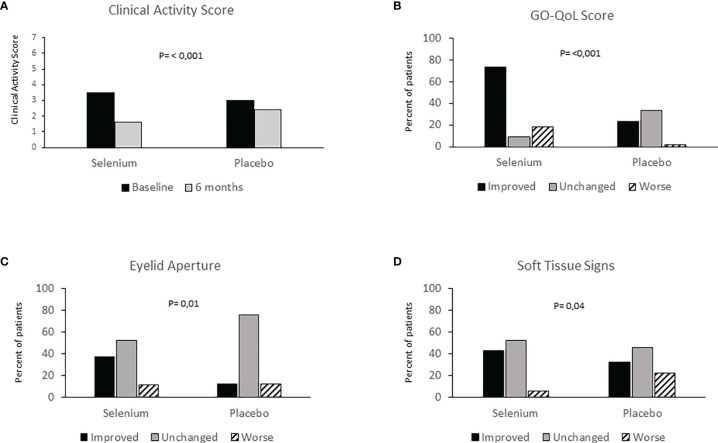
**(A)** Clinical Activity Score (CAS) evaluation at baseline and 6 months in patients with mild Graves’ Orbitopathy treated with selenium or placebo; **(B)** Graves’ orbitopathy-specific quality-of-life questionnaire (GO-QOL) at baseline and 6 months in patients with mild Graves’ Orbitopathy treated with selenium, placebo or Pentoxifylline; **(C)** Eyelid aperture at baseline and 6 months in patients with mild Graves’ Orbitopathy treated with selenium, placebo or Pentoxifylline; **(D)** Soft tissue signs at baseline and 6 months in patients with mild Graves’ Orbitopathy treated with selenium, placebo or Pentoxifylline. Redrawn from the Engl J Med 2011; 364: 1920-1931.

The first clinical, pilot study on antioxidants, showed an improvement of GO soft tissue involvement in patients treated with an antioxidant mixture containing allopurinol plus nicotinamide, compared with those given placebo (54). After these promising results, the European Group on Graves’ Orbitopathy (EUGOGO) performed a randomized, placebo-controlled, multicenter, clinical trial in European countries known to have marginal selenium-deficiency, to investigate the effect of selenium and pentoxifylline in mild GO (4). One hundred and fifty-nine patients with mild GO were randomized to receive sodium selenite (100 mcg twice/day, equivalent to 91.3 μg of selenium), pentoxifylline (600 mg twice/day), or placebo (twice/day) for 6 months, followed by a follow-up period of 6 months. The overall eye outcome—assessed at the end of treatment by a change in a composite score including exophthalmometry, clinical activity score (CAS), measurement of eyelid aperture, diplopia and visual acuity—as well as the quality of life were significantly better in the selenium group. GO improved in 61% of patients treated with selenium and in 36% of patients receiving placebo, whereas the eye disease worsened in 7 and 26% of patients in the selenium and placebo group, respectively (**Figure 2**). The effect of pentoxifylline was not relevant compared to placebo. Similarly, at 6 months, the scores of the quality of life of GO (GO-QOL) increased from baseline by 6 or more points for visual functioning in 33 patients (62%) and for appearance in 40 patients (75%). Interestingly, the majority of selenium-treated patients who had a positive change in eyelid aperture, soft tissue involvement, or both, also had an improvement of 6 points or more on the appearance subscale of GO-QOL [84%; 95% confidence interval (CI), 67 to 95] and on visual functioning subscale (72%; 95% CI, 53 to 86), as well as in the overall score (81%; 95% CI 63 to 93). The GO improvement following treatment with selenium was maintained also at 12 months, suggesting that the beneficial effect of selenium is persistent after treatment withdrawal. A limit of this study is that neither baseline not end of treatment selenium concentrations were not measured. Therefore, it remains unclear whether correction of selenium deficiency accounted for the beneficial effects of selenium administration.

## Conclusions

The redox state is a key point in cellular homeostasis and its unbalance leads to the disruption of intracellular reactions, thereby damaging cell structures. Oxidative stress plays an important role in both GH and GO but the question of whether the use of selenium in the management of patients with GH and GO can be truly effective is still to be clarified. Studies performed so far regarding the use of antioxidant agents, especially as selenium, provided conflicting results on the possibility of a new therapeutic approach. The real effectiveness of selenium in patients with GH is very dubious and at this moment there is not enough evidence to use it. Current evidence on selenium supplementation in patients with mild GO provided promising results, suggesting that selenium may improve the quality of life and the course of GO and opening to the possibility of its clinical use. It is also possible that selenium may have beneficial effects in moderate-to-severe GO, especially when patients are selenium-deficient. The action of selenium for the long-term outcome remains to be well cleared. Further clinical studies are needed to define these issues.

## Author Contributions

GL wrote the manuscript. CM and MM contributed to the conception of the manuscript and revised the paper critically. All authors contributed to the article and approved the submitted version.

## Conflict of Interest

The authors declare that the research was conducted in the absence of any commercial or financial relationships that could be construed as a potential conflict of interest.

## References

[1] Hegedüs L Bonnema SJ Winther KH Selenium in the Treatment of Thyroid Diseases: An Element in Search of the Relevant Indications? Eur Thyroid J (2016) 5:149–51. 10.1159/000448002 PMC5091242 27843804

[2] Calissendorff J Mikulski E Larsen EH Möller M A Prospective Investigation of Graves’ Disease and Selenium: Thyroid Hormones, Auto-Antibodies and Self-Rated Symptoms. Eur Thyroid J (2015) 2:93–8. 10.1159/000381768 PMC4521074 26279994

[3] Leo M Bartalena L Rotondo Dottore G Piantanida E Premoli P Ionni I Effects of selenium on short-term control of hyperthyroidism due to Graves’ disease treated with methimazole: Results of a randomized clinical trial. J Endocrinol Invest (2017) 40:281–7. 10.1007/s40618-016-0559-9 27734319

[4] Marcocci C Kahaly GJ Krassas GE Bartalena L Prummel M Stahl M European Group on Graves’ Orbitopathy. Selenium and the Course of Mild Graves’ Orbitopathy. N Engl J Med (2011) 364:1920–31. 10.1056/NEJMoa1012985 21591944

[5] Köhrle J Selenium and the thyroid. Curr Opin Endocrinol Diabetes Obes (2015) 22:392–401. 10.1097/MED.0000000000000190 26313901

[6] Esposito D Rotondi M Accardo G Vallone G Conzo G Docimo G Influence of short-term selenium supplementation on the natural course of Hashimoto’s thyroiditis: Clinical results of a blinded placebo-controlled randomized prospective trial. J Endocrinol Invest (2017) 40:83–9. 10.1007/s40618-016-0535-4 27572248

[7] De Farias CR Cardoso BR de Oliveira GM de Mello Guazzelli IC Catarino RM Chammas MC A randomized-controlled, double-blind study of the impact of selenium supplementation on thyroid autoimmunity and inflammation with focus on the GPx1 genotypes. J Endocrinol Invest (2015) 38:1065–74. 10.1007/s40618-015-0285-8 25894865

[8] Leporati P Groppelli G Zerbini F Rotondi M Chiovato L Etiopathogenesis of Basedow’s disease. Trends and current aspects. Nuklearmedizin (2015) 54:204–10. 10.3413/Nukmed-0739-15-04 26293122

[9] Marinò M Latrofa F Menconi F Chiovato L Vitti P Role of genetic and non-genetic factors in the etiology of Graves’ disease. J Endocrinol Invest (2015) 38:283–94. 10.1007/s40618-014-0214-2 25421156

[10] Komosinska-Vassev K Olczyk K Kucharz EJ Marcisz C Winsz-Szczotka K Kotulska A Free radical activity and antioxidant defense mechanisms in patients with hyperthyroidism due to Graves’ disease during therapy. Clin Chim Acta (2000) 300:7–117. 10.1016/S0009-8981(00)00306-5 10958867

[11] Duntas LH Boutsiadis A Tsakris A Impaired Metabolism of Selenome-thionine in Graves’ Disease: A Biokinetics Study of Soft Gel Capsule Formulation. Horm Metab Res (2017) 49:589–94. 10.1055/s-0043-113573 28679141

[12] Wilson R Chopra M Bradley H McKillop JH Smith WE Thomson JA Free radicals and Graves’ disease: the effects of therapy. Clin Endocrinol (Oxf) (1989) 30:429–33. 10.1111/j.1365-2265.1989.tb00442.x 2598476

[13] Smith TJ Hegedus L Graves’ Disease. N Engl J Med (2016) 375:1552–65. 10.1056/NEJMra1510030 27797318

[14] Menconi F Marcocci C Marinò M Diagnosis and classification of Graves’ disease. Autoimmun Rev (2014) 13:398–402. 10.1016/j.autrev.2014.01.013 24424182

[15] Bartalena L Fatourechi V Extrathyroidal manifestations of Graves’ disease: a 2014 update. J Endocrinol Invest (2014) 37:691–700. 10.1007/s40618-014-0097-2 24913238

[16] Papi G Corsello MS Pontecorvi A Clinical concepts on thyroid emergencies. Front Endocrinol (2014) 5. article 102. 10.3389/fendo.2014.00102 PMC4076793 25071718

[17] Marinò M Latrofa F Menconi F Chiovato L Vitti P An update on the medical treatment of Graves’ hyperthyroidism. J Endocrinol Invest (2014) 37:1041–8. 10.1007/s40618-014-0136-z 25185644

[18] Lanzolla G Vannucchi G Ionni I Campi I Sikahaly F Lazzaroni E Cholesterol serum levels and use of statins in Graves’ Orbitopathy: A new starting point for the therapy. Front Endocrinol (2020) 10:933. 10.3389/fendo.2019.00933 PMC6987298 32038490

[19] Halliwell B Gutteridge JM Lipid peroxidation, oxigen radicals, cell damage, and antioxidante therapy. Lancet (1984) 1:1396–7. 10.1016/S0140-6736(84)91886-5 6145845

[20] Valko M Leibfritz D Moncol J. Cronin MT Mazur M Telser J Free radicals and antioxidants in normal physiological function and human disease. Int J Biochem Cell Biol (2007) 39:44–84. 10.1016/j.biocel.2006.07.001 16978905

[21] Halliwell B Gutteridge JMC Cellular responses to oxidative stress: adaption, damage, repair, senescence and death. Free Radicals Biol Med (2007) 376(18):1748–61. 10.1056/NEJMoa1614949

[22] Venditti P Balestrini M Di Meo S De Leo T Effect of thyroid state on lipid peroxidation, antioxidant defenses, and susceptibility to oxidative stress in rat tissues. J Endocrinol (1997) 155:151–7. 10.1677/joe.0.1550151 9390017

[23] Yamada T Mishima T Sakamoto M Sugiyama M Matsunaga S Wada M Oxidation of myosin heavy chain and reduction in force production in hyperthyroid rat soleus. J Appl Physiol (2006) 100:1520–6. 10.1152/japplphysiol.01456.2005 16397059

[24] Miot F Van Sande J Many MC Dumont JE Roles of hydrogen peroxide in thyroid physiology and disease. J Clin Endocrinol Metab (2007) 92:3764–73. 10.1210/jc.2007-0660 17666482

[25] Asayama K Dobashi H Hayashibe H Megata Y Kato K Lipid peroxidation and free radical scavengers in thyroid dysfunction in the rat: a possible mechanism of injury to hearth and skeletal muscle in hyperthyroidism. Endocrinology (1987) 121:2112–8. 10.1210/endo-121-6-2112 2824181

[26] Chehade J Kim J Pinas JL Mooradian AD Age-related changes in the thyroid hormone effects on malondialdehyde modified proteins in the rat heart. Proc Soc Exp Biol Med (1999) 222:59–64. 10.1111/j.1525-1373.1999.09995.x 10510246

[27] Asayama K Dobashi K Hayashibe H Kato K Vitamin E protects against thyroxine-induced acceleration of lipid peroxidation in cardiac and skeletal muscles in rats. J Nutr Sci Vitaminol (Tokyo) (1989) 35:407–18. 10.3177/jnsv.35.407 2632676

[28] Abalovich M Llesuy S Gutierrez S Repetto M Peripheral parameters of oxidative stress in Graves disease: the effect of methimazole and 131 I iodine treatment. Clin Endocrinol (Oxf) (2003) 59:321–7. 10.1046/j.1365-2265.2003.01850.x 12919155

[29] Bednarek J Wysocki H Sowiński J Oxidative stress peripheral parameters in Graves’ disease: the effect of methimazole treatment in patients with and without infiltrative ophthalmopathy. Clin Biochem (2005) 38:13–8. 10.1016/j.clinbiochem.2004.09.015 15607311

[30] Rybus-Kalinowska B Zwirska-Korczala K Kalinowski M Kukla M Birkner E Jochem J Activity of antioxidative enzymes and concentration of malondialdehyde as oxidative status markers in women with newly diagnosed Graves-Basedow disease and after thiamazole therapy leading to euthyroidism. Pol Arch Med Wewn (2008) 118:420–5. 10.20452/pamw.438 18714737

[31] Cetinkaya A Kurutas EB Buyukbese MA Kantarceken B Bulbuloglu E Levels of malondialdehyde and superoxide dismutase in subclinical hyperthyroidism. Mediators Inflammation (2005) 59(3):321–7. 10.1155/MI.2005.57 PMC1513061 15770068

[32] Weetman AP Effect of the anti-thyroid drug methimazole on interleukin-1 and interleukin- 2 levels in vitro. Clin Endocrinol (Oxf) (1986) 25:133–42. 10.1111/j.1365-2265.1986.tb01674.x 3098461

[33] Aslan M Cosar N Celik H Aksoy N Dulger AC Begenik H Evaluation of oxidative status in patients with hyperthyroidism. Endocrine (2011) 40:285–29. 10.1007/s12020-011-9472-3 21519910

[34] Bartalena L Tanda ML Piantanida E Lai A Oxidative stress and Graves’ ophthalmopathy: in vitro studies and therapeutic implications. Biofactors (2003) 19:155–63. 10.1002/biof.5520190308 14757966

[35] Marcocci C Leo M Altea MA Oxidative stress in Graves’ disease. Eur Thyroid J (2012) 2:80–7. 10.1159/000337976 PMC3821469 24783001

[36] Diana T Daiber A Oelze M Neumann S Olivo PD Kanitz M Stimulatory TSH-Receptor antibodies and oxidative stress in Graves’ Disease. JCEM (2018) 103:3668–77. 10.1210/jc.2018-00509 PMC6179174 30099546

[37] Heufelder AE Wenzel BE Bahn RS Methimazole and propylthiouracil inhibit the oxygen free radical-induced expression of a 72 kilodalton heat shock protein in Graves’ retroocular fibroblasts. J Clin Endocrinol Metab (1992) 74:737–42. 10.1002/biof.5520190308 1532179

[38] Rotondo Dottore G Leo M Casini G Latrofa F Cestari L Sellari-Franceschini S Anti-oxidant actions of selenium in orbital fibroblasts: a basis for the effects of selenium in Graves’ orbitopathy. Thyroid (2016) 27:271–8. 10.1089/thy.2016.0397 27824294

[39] Burch HB Lahiri S Bahn RS Barnes S Superoxide radical production stimulates retroocular fibroblast proliferation in Graves’ ophthalmopathy. Exp Eye Res (1997) 65:311–6. 10.1006/exer.1997.0353 9268599

[40] Lu R Wang P Wartofsky L Sutton BD Zweier JL Bahn RS Oxygen free radicals in interleukin-1beta-induced glycosaminoglycan production by retro-ocular fibroblasts from normal subjects and Graves’ ophthalmopathy patients. Thyroid (1999) 9:297–303. 10.1089/thy.1999.9.297 10211608

[41] Hondur A Konuk O Dincel AS Bilgihan A Unal M Hasanreisoglu B Oxidative stress and antioxidant activity in orbital fibroadipose tissue in Graves’ ophthalmopathy. Curr Eye Res (2008) 33:421–7. 10.1080/02713680802123532 18568878

[42] Tsai CC Wu SB Cheng CY Kao SC Kau HC Chiou SH Increased oxidative DNA damage, lipid peroxidation, and reactive oxygen species in cultured orbital fibroblasts from patients with Graves’ ophthalmopathy: evidence that oxidative stress has a role in this disorder. Eye (Lond) (2010) 24:1520–5. 10.1038/eye.2010.31 20300129

[43] Tsai CC Wu SB Cheng CY Kao SC Kau HC Lee SM Increased response to oxidative stress challenge in Graves’ ophthalmopathy orbital fibroblasts. Mol Vis (2011) 17:2782–8. 10.1006/exer.1997.0353 PMC3209425 22065933

[44] Tsai CC Kao SC Cheng CY Kau HC Hsu WM Lee CF Oxidative stress change by systemic corticosteroid treatment among patients having active graves ophthalmopathy. Arch Ophthalmol (2007) 125:1652–6. 10.1001/archopht.125.12.1652 18071117

[45] Wiersinga WM Smoking and thyroid. Clin Endocrinol (Oxf) (2013) 79:145–51. 10.1111/cen.12222 23581474

[46] Akarsu E Buyukhatipoglu H Aktaran S Kurtul N Effects of pulse methylprednisolone and oral methylprednisolone treatments on serum levels of oxidative stress markers in Graves’ ophthalmopathy. Clin Endocrinol (Oxf) (2011) 74:118–24. 10.1111/j.1365-2265.2010.03904.x 21044110

[47] Rayman MP The importance of selenium to human health. Lancet (2000) 356:233–41. 10.1016/S0140-6736(00)02490-9 10963212

[48] Visser TJ Regulation of thyroid function, synthesis, and function of thyroid hormones. Thyroid Dis (2018) 125(12):1652–6. 10.1007/978-3-319-45013-1_1

[49] Drutel A Archambeaud F Caron P Selenium and the thyroid gland. Clin Endocrinol (2013) 78:155–64. 10.1111/cen.12066 23046013

[50] Rayman MP Selenium and human health. Lancet (2012) 379:1256–68. 10.1016/S0140-6736(11)61452-9 22381456

[51] Duntas LH Selenium and Inflammation: Underlying Anti-inflammatory Mechanisms. Horm Metab Res (2009) 41:443–44. 10.1055/s-0029-1220724 19418416

[52] Duntas LH Selenium and the thyroid: a close-knit connection. J Clin Endocrinol Metab (2010) 95:5180–8. 10.1210/jc.2010-0191 20810577

[53] Stranges S Marshall JR Natarajan R Donahue RP Trevisan M Combs GF Effects of long term supplementation on the incidence of type 2 diabetes: a randomized trial. Ann Int Med (2007) 147:217–22. 10.7326/0003-4819-147-4-200708210-00175 17620655

[54] Marinò M Marcocci C Vitti P Chiovato L Bartalena L Selenium in the treatment of thyroid diseases. Eur Thyroid J (2017) 6:113–4. 10.1159/000456660 PMC5422754 28589094

[55] Vrca VB Mayer L Skreb F Rahelić D Marušić S Antioxidant supplementation and serum lipids in patients with Graves’ disease: effect on LDL-cholesterol. Acta Pharm (2012) 62:115–22. 10.2478/v10007-012-0005-2 22472454

[56] Kahaly GJ Riedl M König J Diana T Schomburg L Double-Blind, Placebo-Controlled, Randomized Trial of Selenium in Graves Hyperthyroidism. J Clin Endocrinol Metab (2017) 102:4333–41. 10.1210/jc.2017-01736 29092078

[57] Guerra LN Ríos de Molina Mdel C Miler EA Moiguer S Karner M Burdman JA Antioxidants and methimazole in the treatment of Graves’ disease: effect on urinary malondialdehyde levels. Clin Chim Acta (2015) 352:115–20. 10.1016/j.cccn.2004.08.020 15653105

[58] Bartalena L Baldeschi L Boboridis K Eckstein A Kahaly GJ Marcocci C European Group on Graves’ Orbitopathy (EUGOGO). The 2016 European Thyroid Association/European Group on Graves’ Orbitopathy Guidelines for the Management of Graves’ Orbitopathy. Eur Thyroid J (2016) 5:9–26. 10.1159/000443828 27099835 PMC4836120

[59] Stan MN Salvi M Management of endocrine disease: rituximab therapy for Graves’ orbitopathy - lessons from randomized control trials. Eur J Endocrinol (2016) 176:R101–9. 10.1530/EJE-16-0552 27760790

[60] Douglas RS Kahaly GJ Patel A Sile S Thompson EHZ Perdok R Teprotumumab for the Treatment of Active Thyroid Eye Disease. N Engl J Med (2020) 382(4):341–52. 10.1056/NEJMoa1910434 31971679

[61] Smith TJ Kahaly GJ Ezra DG Fleming JC Dailey RA Tang RA Teprotumumab for thyroid-associated ophthalmopathy. N Engl J Med (2017) 376:1748–61. 10.1056/NEJMoa1614949 PMC5718164 28467880

[62] Kahaly GJ Riedl M König J Pitz S Ponto K Diana T Combined mycophenolate + prednisolone therapy is more effective than prednisolone in active and moderate-to-severe Graves’ Orbitopathy – a randomized, observer blind, multicenter trial. Lancet Diabetes Endocrinol (2018) 6:287–98. 10.1530/EJE-16-0552 29396246

[63] Pérez-Moreiras JV Treatment of active corticosteroid-resistant Graves’ Orbitopathy. Ophthalmic Plast Reconstr Surg (2014) 30:162–7. 10.1097/IOP.0000000000000037 24503568

[64] Sisti E Coco B Menconi F Leo M Rocchi R Latrofa F Intravenous glucocorticoid therapy for Graves’ ophthalmopathy and acute liver damage: an epidemiological study. Eur J Endocrinol (2015) 172:269–76. 10.1530/EJE-14-0712 25661744

[65] Sisti E Coco B Menconi F Leo M Rocchi R Latrofa F Age and Dose Are Major Risk Factors for Liver Damage Associated with Intravenous Glucocorticoid Pulse Therapy for Graves; Orbitopathy (2015). Thyroid 25:846–50. 10.1089/thy.2015.0061 26090805

[66] Marcocci C Watt T Altea MA Rasmussen AK Feldt-Rasmussen U Orgiazzi J Fatal and non-fatal adverse events of glucocorticoid therapy for Graves’ orbitopathy: a questionnaire survey among members of the European Thyroid Association. Eur J Endocrinol (2012) 166:247–53. 10.1530/EJE-11-0779 22058081

[67] Rotondo Dottore G Chiarini R De Gregorio M Leo M Casini G Cestari L Selenium rescues orbital fibroblasts from cell death induced by hydrogen peroxide: another molecular basis for the effects of selenium in Graves’ orbitopathy. Endocrine (2017) 58:386–9. 10.1007/s12020-016-1226-9 28097622

[68] Tsai CC Wu SB Kao SC Kau HC Lee FL Wei YH The protective effect of antioxidants on orbital fibroblasts from patients with Graves’ ophthalmopathy in response to oxidative stress. Mol Vis (2013) 19:927–34. 10.1007/s12020-017-1255-z PMC3654843 23687429

